# Is long-term serum preservation suitable for research studies? Effect of time and temperature on the measurement of anti-*Leishmania* antibodies in canine sera samples

**DOI:** 10.1080/01652176.2025.2532396

**Published:** 2025-07-15

**Authors:** Diana Marteles, María Eugenia Lebrero, Sergio Villanueva-Saz, Clara Esteban Sanz, Víctor Martín, Antonio Fernández, Pablo Quilez, Maite Verde, Patricia Galan-Malo, M. Dolores Pérez

**Affiliations:** a Clinical Immunology Laboratory, Veterinary Faculty, University of Zaragoza, Zaragoza, Spain; b Department of Animal Pathology, Veterinary Faculty, University of Zaragoza, Zaragoza, Spain; c Instituto Agroalimentario de Aragón-IA2 (Universidad de Zaragoza-CITA), Zaragoza, Spain; d Department of Animal Production and Sciences of the Food, Veterinary Faculty, University of Zaragoza, Zaragoza, Spain; e ZEULAB, S.L. C/Bari 25 dpdo, Zaragoza

**Keywords:** Antibodies, dog, immunoglobulins, *Leishmania infantum*, long-term, stability

## Abstract

The stability of immunoglobulin G (IgG) antibodies is critical for diagnostic and research applications in veterinary medicine. This study evaluated the long-term stability of anti-*Leishmania infantum* IgG in canine serum samples under different storage conditions (−20 °C and −80 °C) over 2.5 years. Fifty-six serum samples were classified based on antibody concentration into low, medium, and high positive groups using an in-house enzyme-linked immunosorbent assay. Each sample was divided into aliquots and analyzed after different storage times (6 months, 1 year, 1.5, and 2.5 years). No statistically significant differences were observed in IgG concentrations across storage durations or between storage temperatures. Median antibody levels remained consistent, with minor variations attributed to assay-related variability. Correlation analyses showed strong agreement between initial and final measurements (R^2^ = 0.859 at −20 °C, R^2^ = 0.957 at −80 °C). The study underscores the suitability of −20 °C and −80 °C storage for preserving anti-*Leishmania* antibodies, providing valuable insights for serological diagnostics and research in veterinary science. Proper sample handling and aliquoting are recommended to maintain antibody integrity in routine diagnostics and long-term studies.

## 1. Introduction

The evolution of humoral immunity research has been closely associated with major breakthroughs in medicine, particularly in the prevention and management of infectious diseases in both humans and animals (Day 2012; Playfair and Bancroft 2013). In veterinary medicine, immunoglobulin G (IgG) is especially significant due to its abundance in serum and its critical role in diagnostic applications (Peters 2016).

Laboratory diagnostic plays an essential role in veterinary practice, particularly in identifying and managing diseases in companion animals such as dogs. Among these diagnostic tools, serum biochemistry is particularly prominent, valued not only for its clinical significance but also for its simplicity and routine integration into veterinary clinical laboratories. This widespread use underscores its importance in meeting the growing demands of small animal clinics (Aguilar-Montes de Oca et al. 2022).

Although banked serum specimens subjected to repeated freeze-thaw cycles are commonly used, there is a lack of comprehensive data on how prolonged storage impacts antibody measurement. This gap is particularly relevant for highly sensitive assays like enzyme-linked immunosorbent assays (ELISAs), where structural changes of antibodies during storage could compromise the accuracy of results (Correia, 2010; Yen et al. 2024).

In human medicine, extensive research has explored the stability of IgG under various laboratory conditions, such as fluctuating storage temperatures (Solberg et al. 2024), prolonged storage times (Castejon et al. 2014), and repeated freezing-thawing processes (Pinsky et al. 2003). Additionally, the choice of assay significantly influences antibody stability (Kanji et al. 2021; Solberg et al. 2024). However, similar studies evaluating antibody stability, including IgG, under different conditions are sparse in veterinary medicine.

In the context of canine leishmaniosis, serological testing remains the primary method for diagnostic confirmation, with quantitative techniques such as the indirect immunofluorescent antibody test (IFAT) and enzyme-linked immunosorbent assay (ELISA) being the most widely used (Solano-Gallego et al. 2011). These methods differ mainly in their use of antigens and technical procedures. For instance, IFAT employs whole parasites immobilized on a slide and relies on fluorescence microscopy for evaluation, while ELISA uses various antigen types in plastic wells and quantifies results through absorbance measurements (Villanueva-Saz et al. 2022). These serological tools are indispensable for detecting anti-*Leishmania* antibodies, supporting applications such as seroepidemiological research, diagnostic method comparisons, clinical trials, and immune response studies.

Recent findings suggest that seasonal variations in anti-*Leishmania infantum* antibody titers in dogs are crucial for designing effective clinical trials to assess therapeutic and preventive strategies against canine leishmaniosis (Cavalera et al. 2024). In laboratory research, serum sample storage is a fundamental aspect for many studies, particularly those focusing on seroepidemiological surveys across diverse populations and circumstances. Nevertheless, research on the stability of antibodies under different laboratory conditions, including storage duration and temperature, remains limited in veterinary science.

This study aimed to fill this gap by evaluating the stability of IgG against *L. infantum* under varying storage conditions including temperature and time.

## 2. Material and methods

### 2.1. Samples

Fifty-six fresh serum samples were selected from the sera collection of Clinical Pathology Laboratory (Universidad de Zaragoza, Spain). Samples were received in the laboratory from several veterinary clinics for different diagnostic purposes: annual screening program for clinically healthy dogs, cases of suspected clinical leishmaniosis, blood donor screening program and pre-vaccination screening for *L. infantum* infection. These samples were collected from 1^st^ March 2022 to 11^th^ March 2022. For this study, samples with different anti-*Leishmania* IgG status were included which were classified as low positive (*n* = 22), medium positive (*n* = 14) and high positive (*n* = 20) based on in-house ELISA test (reference). Sera was classified as high positive when the Optical Density (O.D.) was equal or higher than 2.000 (≥2.000), medium positive when the O.D. ranged between 1.000 and 2.000 and, low positive when the O.D. ranged between 0,210 and 1.000. Clinical Pathology Laboratory received sera anonymized without any personal information.

Serum from animal was divided into eight aliquots of 200 µL each, four aliquots were stored at −20 °C and four at −80 °C. For each freezing temperature, samples were thawed once time at different time points (6 months, 1 year, 1.5 years; 2.5 years).

### 2.2. Detection of L. infantum antibodies by a quantitative ELISA

Humoral analysis of IgG concentration was performed using 96-well plates. For the in-house ELISA, the fresh crude antigen (strain MHOM/FR/78/LEM 75 belonging to *Leishmania infantum* zimodeme MON-1) whose concentration was determined by the BCA assay, was adjusted to a concentration of 20 µg/mL with sterile commercial phosphate buffered saline (PBS) (Thermo Fisher Scientific, Waltham, Massachusetts, USA). Briefly, each plate was coated with 100 µL/well of the 20 µg/mL fresh antigen solution in 0.1 M carbonate/bicarbonate buffer (pH 9.6) and incubated overnight at 4 °C. Then, a volume of 100 µl of dog serum, diluted 1:800 in PBS containing 0.05% Tween 20 (PBST) and 1% dry skimmed milk (PBST-M) were added to each well. The plates were incubated for 1 h (h) at 37 °C in a moist chamber. After washing the plates three times with PBST for 3 min (min) followed by one wash with PBS for 1 min, 100 µL of Protein A conjugated to horseradish peroxidase (Thermo Fisher Scientific, Waltham, Massachusetts, USA) diluted 1:20000 in PBST-M was added to each well. The plates were incubated for 1 h at 37 °C in a moist chamber, followed by washes with PBST and PBS as described above. The substrate solution (ortho-phenylene-diamine) dissolved-diluted in stable peroxide substrate buffer (Thermo Fisher Scientific, Waltham, Massachusetts, USA) were added (100 µL per well) and incubated for 20 ± 5 min at room temperature in the dark. The enzymatic reaction was stopped by adding 100 µL of 2.5 M H_2_SO_4_ to each well. Absorbance values were read at 492 nm (reference wavelength) in an automatic microELISA reader (ELISA Reader Labsystems Multiskan, Midland, Canada). A calibration curve was obtained for each assay using purified dog IgG (Bio-Rad Laboratories Inc.) standards within a concentration ranging between 0.1 to 1.5 µg/mL. The standard curve for IgG was calculated using a computer generated second degree polynomic curve. Plates were repeated when R^2^-value of standard curve was below 0.98. All samples and standards were analyzed in duplicate on each plate.

### 2.3. Statistical analysis

Statistical analysis was performed using the IBM^®^ SPSS^®^ Statistics software version 29 (SPSS Inc. Chicago, IL, USA). A p-value < 0.05 was considered as statistically significant. As IgG concentration values within frozen storage conditions were not normally distributed (Shapiro-Wilk test, *p* < 0.000) a descriptive study of the concentration IgG on day zero, 6 months, 1 year, 1.5 and 2.5 years was performed, and the medians were compared using a Wilcoxon Signed Rank test. The difference in IgG concentrations between storage conditions (-20° versus −80 °C) for each time point were studied using the Mann-Whitney U test. Additionally, calculation of the percent difference (%difference) between the initial analysis result (baseline, day zero) and the result measured at different storage times (6 months, 1 year, 1.5 years; 2.5 years) was done using the following equation:

$$\%\text{difference}=\frac{\mathrm{Concentration}\;\text{after}\;\text{strage}-\text{concentrationa}\;\text{at}\;\text{day}\;\text{zero}}{\text{concentration}\;\text{at}\;\text{day}\;\text{zero}}\times 100$$

Additionally, percent differences between each time point and the measurement of day zero obtained were compared using the Friedman test.

Based on a Type I error (α) of 5% (i.e. 95% confidence) and a Type II error (β) of 20% (i.e. 80% power), a minimum detectable difference of 20% was assumed. Accordingly, the sample size calculation using day-zero data indicated that at least 20 animals were required to detect this 20% difference. When stratified by seropositivity groups, at least 20 low-positive, 14 medium-positive, and 20 high-positive samples were needed to detect the minimal difference. Finally, we determined the median and interquartile range for antibody concentrations at each observation point, as well as performed correlation and regression analyses. All statistical analyses were conducted using MedCalc^®^ Statistical Software version 20.118 (Ostend, Belgium; https://www.medcalc.org; 2022).

## 3. Results

A number of 56 serum samples were collected from dogs for different diagnostic purposes: 14 for annual screening program, 33 for cases of suspected clinical leishmaniosis, 4 blood donor screening program and finally 5 dogs for pre vaccination screening. A number of 27 out of the dogs were males and 29 females (43%). Finally, 35 were pure-breed and 31 mixed-breed.

At the day zero, the median concentration ± interquartile range (median ± IQR) of antibodies from all animals was 0.396 ± 0.716 µg/mL and similar results were obtained at the different time points of storage at −20 °C (6 months, 0.408 ± 0.703 µg/mL; 1 year, 0.394 ± 0.699 µg/mL, 1.5 years, 0.351 ± 0.661 µg/mL; and 2.5 years, 0.368 ± 0.708 µg/mL) at −20 °C. Similarly, the median was similar at −80 °C in the different storage times (6 months, 0.366 ± 0.713 µg/mL; 1 year, 0.392 ± 0.692 µg/mL; 1.5 years, 0.383 ± 0.635 µg/mL; and 2.5 years, 0.352 ± 0.703 µg/mL).

In Table 1, the median ± IQR concentration level of antibodies is described for each seropositive status determined as low positive, medium positive and high positive.

**Table 1.** Values of the Median ± IQR corresponding to the concentration of IgG anti-*Leishmania* obtained in each serological status groups during the different time points.

Serological status	Concentration of IgG anti-*Leishmania* (µg/mL)								
	Zero days	6 months		1 year		1.5 years		2.5 years	
		−20 °C	−80 °C	−20 °C	−80 °C	−20 °C	−80 °C	−20 °C	−80 °C
Low positive	0.17 ± 0.08	0.18 ± 0.10	0.17 ± 0.11	0.17 ± 0.07	0.17 ± 0.11	0.16 ± 0.07	0.19 ± 0.09	0.17 ± 0.10	0.18 ± 0.09
Medium positive	0.43 ± 0.17	0.41 ± 0.17	0.42 ± 0.21	0.46 ± 0.19	0.44 ± 0.13	0.36 ± 0.11	0.40 ± 0.14	0.44 ± 0.18	038 ± 0.11
High positive	1.02 ± 0.77	1.16 ± 0.67	1.10 ± 0.65	1.07 ± 0.66	1.02 ± 0.76	0.97 ± 0.85	0.99 ± 0.68	1.48 ± 0.83	1.03 ± 0.67

**Figure 1.**
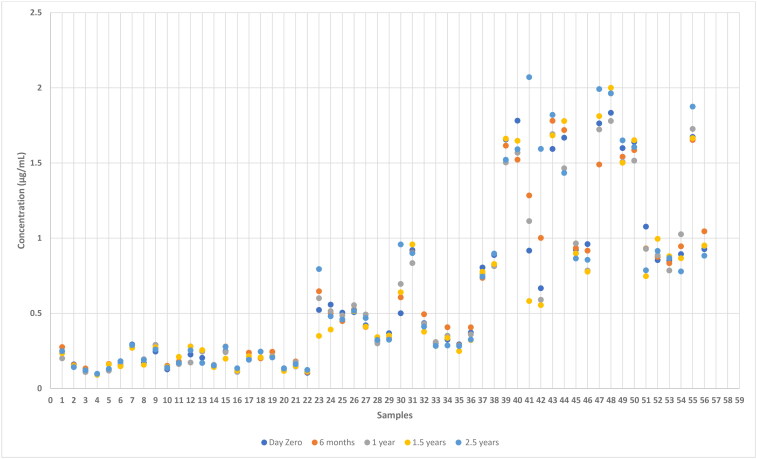
Concentration of anti-*Leishmania* IgG in dog serum samples after storage at −20 °C for different times determined by ELISA.

**Figure 2.**
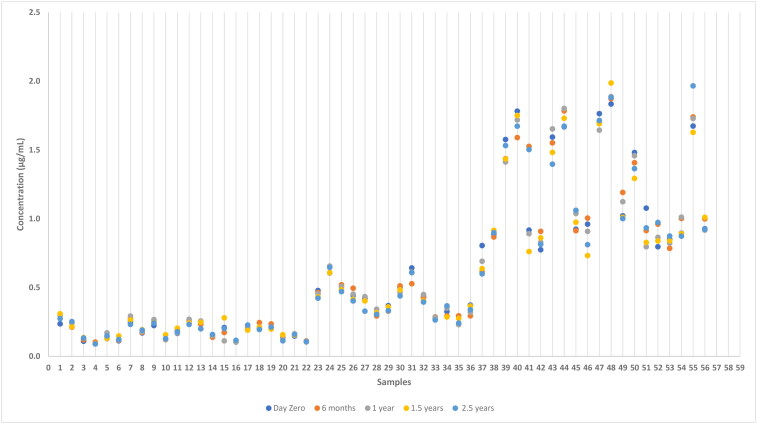
Concentration of anti-*Leishmania* IgG in dog serum samples after storage at −80 °C for different times determined by ELISA.

**Figure 3.**
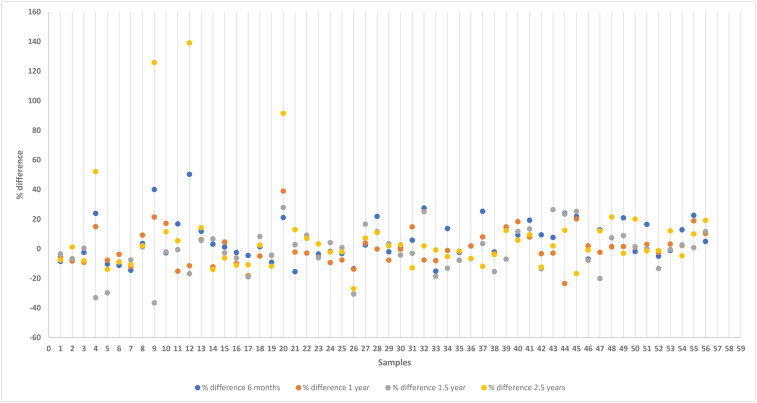
Percent difference between the concentration of anti-*leishmania* IgG at day zero and after storage at −20 °C for 6 months, 1 year, 1.5 and 2.5 years. The %difference calculated is depicted as a function of the measurement at day zero. Results are expressed as percentage respect to day zero (100%).

**Figure 4.**
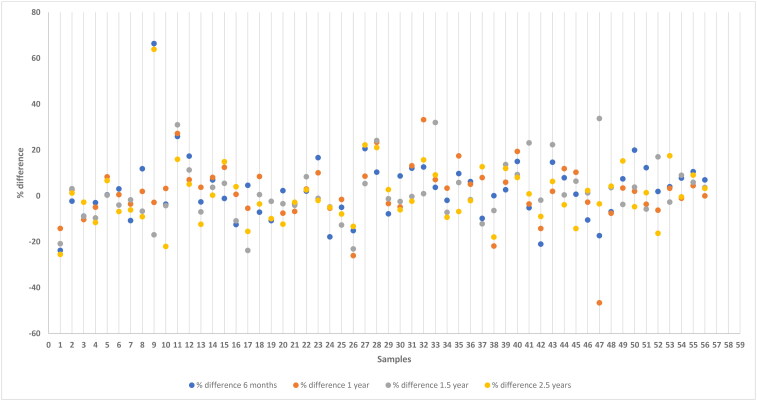
Percent difference between the initial anti-*Leishmania* IgG measure by ELISA and the repeated measure after storage at −80 °C for 6 months, 1 year, 1.5 and 2.5 years. The %difference calculated is depicted as a function of the measurement at day zero. Results are expressed as percentage respect to day zero (100%).

Results from the analysis of samples stored at −20 °C is presented in Figure 1 and samples stored at −80 °C is presented in Figure 2. The stability of anti-*Leishmania* antibodies at −20 °C and −80 °C were examined for the variability between the concentration at day zero, and at six months, 1 year, 1.5 and 2.5 years, respectively, by calculation of %difference as described in the Materials and Methods section. The %difference calculated for each measurement as a function of the concentration at day zero is depicted in Figures 3 and 4, respectively.

**Figure 5.**
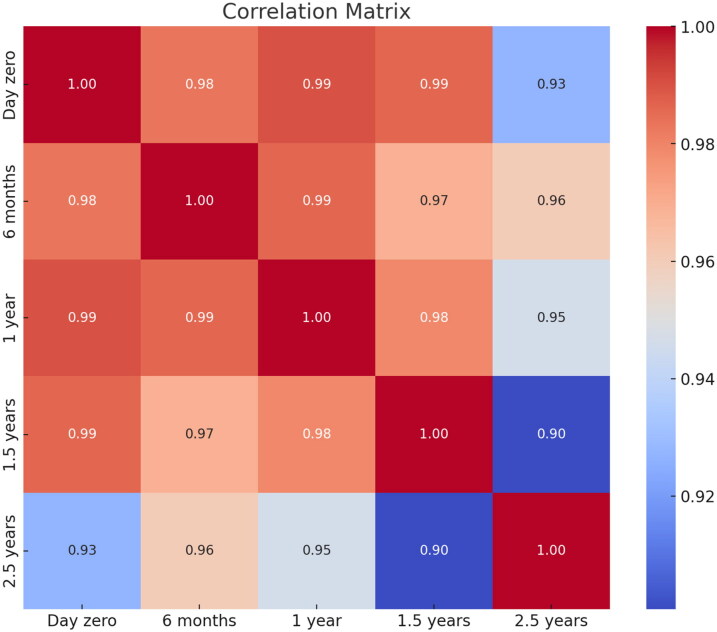
Correlation matrix displaying the pairwise relationships between the variables in the dataset after storage at −20 °C.

**Figure 6.**
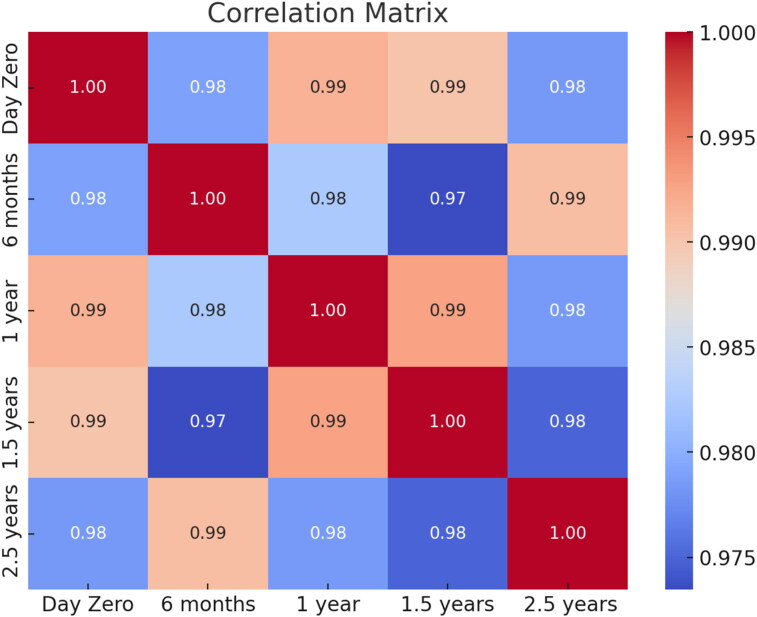
Correlation matrix displaying the pairwise relationships between the variables in the dataset after storage at −80 °C.

In our study, the % difference varied more for the samples classified as low positive compared to those classified as medium or high values for both temperature conditions (Supplementary material). No statistically significant differences were detected between day zero and the remaining storage times related to the level of IgG antibodies concentration against *L. infantum* stored at −20 °C (6 months, *p* = 0.181; 1 year, *p* = 0.257; 1.5 years, *p* = 0.373; 2.5 years, *p* = 0.782) or at −80 °C (6 months, *p* = 0.579; 1 year, *p* = 0.438; 1.5 years, *p* = 0.415; 2.5 years, *p* = 0.195). Likewise, no statistically significant differences were found between the temperature of storage for the different time points included (6 months, *p* = 0.760; 1 year, *p* = 0.942; 1.5 years, *p* = 0.947; 2.5 years, *p* = 0.716). Furthermore, no statistically significant differences were detected between % difference between time points, considering that % difference was calculated between each time point and the measurement of day zero at −20 °C (*p* = 0.165), and at −80 °C (*p* = 0.490). Additionally, a correlation matrix was performed between time points at −20 °C (Figure 5) and −80 °C (Figure 6), showing that the minimum correlation was 0.90 between 1,5 and 2,5 years at −20 °C, whilst, the minimum correlation was 0.97 between 6 months and 1.5 years. The correlation is high in all cases and slightly lower when compared at a 2.5-year time frame, as the blue zone is located at the outermost part of the figure.

**Figure 7.**
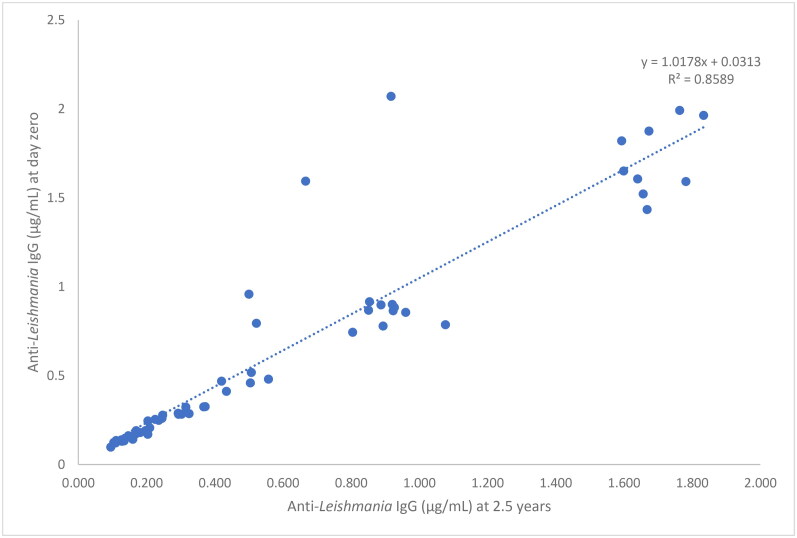
Linear regression of the concentration of anti-*leishmania* IgG (µg/mL) based on long-term (2.5 years) stability data at −20 °C (*n* = 56).

In relation to temperature storage of −20 °C, lineal regression analysis for concentration of all 56 samples between the day zero and the time of long-term stability (2.5 years) was performed. The regression line formula was *y* = 1.018x + 0.031, and the correlation coefficient of 0.859, indicating a strong correlation (Schober et al., 2018) between first analysis and last analysis (Figure 7).

**Figure 8.**
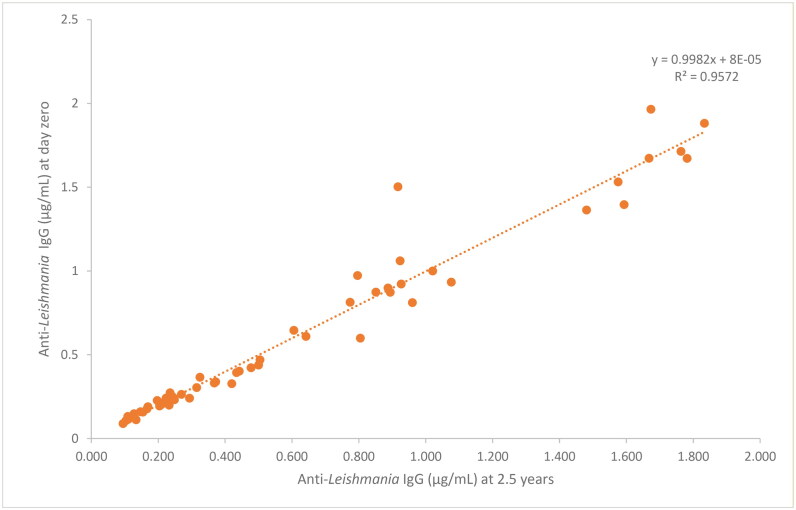
Linear regression of the concentration of anti-*leishmania* IgG (µg/mL) based on long-term (2.5 years) stability data at −80 °C (*n* = 56).

In the case of temperature of storage −80 °C, the lineal regression line formula was *y* = 0.998x + 0.000, and the correlation coefficient of 0.957, indicating also a very strong correlation (Schober et al. 2018), between day zero and 2.5 years (Figure 8).

## 4. Discussion

Results obtained in this study show that the short-term and long-term storage does not affect the stability of anti-*Leishmania* antibodies in canine serum samples after up to 2.5 years at −20 °C and −80 °C storage conditions. Most of the seroepidemiological studies included samples that were stored under variable temperatures from conditions at −18 °C (Bauer et al. 2024) −20 °C (Didkowska et al. 2025), −30 °C (Mahachi et al. 2024), −70 °C (Kanji et al. 2021) or −80 °C (Michaut et al. 2014). Our study supports that long-term frozen storage at 20 °C and 80 °C have a low impact on the concentration of anti-*Leishmania* IgG.

In human medicine, this type of studies is more prevalent and includes IgG against different pathogens. A study demonstrated the stability of anti-human immunodeficiency virus antibodies in serum over an 18-year period when assessed using enzyme immunoassays (Castejon et al. 2014). Other studies which evaluated the stability of anti-immunotherapeutic antibodies in serum samples found a minimal effect of storage conditions at −80 °C for at least 3.5 years and 3–12 freezing–thawing cycles (Michaut et al. 2014). In general, human antibodies in undiluted serum samples remain stable for extended periods (6.3 years) when stored at low freezing temperatures (−20 °C), being not necessary to apply much lower temperatures like −70 °C (Solberg et al. 2024). In our study, the storage period was shorter in comparison with other investigations, however when comparing the temperature of storage, no statistical significant differences were found between −20 °C and 80 °C during the time periods included.

One condition regarding special attention to evaluate the impact of frozen storage on IgG stability is referred to the application of repeated freezing-thawing cycles (Pinsky et al. 2003; Castejon et al. 2017). This condition has shown contradictory results in different studies. Some authors found that freeze-thawing cycles of serum clinical specimens have minimum impact on the stability of IgG antibodies against various pathogens (Castro and Jost 2013; Torelli et al. 2021). Another study observed that multiple freezing-thawing cycles does not affect antibody aggregation reactions (Horn et al. 2019), which were attributed to the phenomenon of protein self-association. Anyway, to prevent the potential negative effect on antibody structure and functionality, it is recommended to divide serum samples into multiple aliquots for long-term storage for better preservation, so only a thawing cycle is performed based on recent review article (Yen et al. 2024).

To our knowledge, in veterinary medicine, limited information related to storage conditions has been published. Recently, a study evaluated the effect of storage time at −20 °C for 30 days and repeated freeze-thaw cycles on reactivity of avian serum IgG against amyloid. This study showed that the concentration of this acute phase protein measured daily was stable. However, more than four freeze-thaw cycles resulted in a significant reduction of serum amyloid A concentration (Rhim et al. 2024).

The potential impact of long-term serum specimen storage on the serodiagnosis of canine vector-borne diseases, such as canine monocytic ehrlichiosis, was recently evaluated using archived samples initially tested for *Ehrlichia canis* IgG antibodies. These samples were stored at −20 °C for a median of 22 years, and the results indicate that serum specimens preserved under these conditions may remain valuable for seroepidemiological surveys assessing exposure to *E. canis* (Karagkouni et al. 2024). In our study, all seropositive samples tested positive in the repeat ELISA performed during the study. However, in the study of *E. canis* IgG antibodies, six samples initially classified as seropositive were found negative in the repeat IFAT test. Several explanations could account for this discrepancy, including differences in the type of antigen coating used on the slides, interlaboratory variations in IFAT technique, damage to the structure and functionality of antibodies due to freezing conditions, particularly in serum samples with low antibody levels and, finally, the possibility of a Type II error (Karagkouni et al. 2024).

Related to the stability of anti-*Leishmania infantum* antibodies in human and canine samples, a study evaluated the storage of freeze-dried sera after storage for 11 months at different temperature conditions including −20 °C and −70 °C. Results of this study showed that that freeze-dried human and dog sera were highly stable under frozen conditions (Kakooei et al. 2014).

Finally, our findings indicate that the storage of biological samples, such as serum, for seroepidemiological studies does not affect the concentration of immunoglobulin G against *L. infantum*. Our study has direct impact on epidemiological implications rather than diagnostic applications, emphasizing the critical role of storage time and conditions.

## 5. Conclusions

IgG antibodies against *L. infantum* were found to maintain stable in serum samples stored at −20 °C and −80 °C up to 2.5 years. Based on our results, the observed low variability is most likely attributable to assay-related variability. These results improve our understanding of the reliability of anti-*Leishmania* antibodies in veterinary health assessments and underscore the importance of proper sample handling in serological diagnostic laboratories. It is expected that results obtained in this study about the stability of IgG against *Leishmania* at different temperatures and times will be extended to IgG obtained against other canine pathogens transmitted by vectors.

## Supplementary Material

Supplementary material_pg.docx

## Data Availability

The datasets supporting the conclusions of this study are included in this article. All analysed data are available from the corresponding author upon request.
