# Supplementary material for: Is long-term serum preservation suitable for research studies? Effect of time and temperature on the measurement of anti-Leishmania antibodies in canine sera samples
Source: Vet Q. 2025 Jul 15;45(1):11–20. doi: 10.1080/01652176.2025.2532396 (PMC12265091; doi:10.1080/01652176.2025.2532396)
Supplement: Supplementary material_pg.docx [file TVEQ_A_2532396_SM6717.docx]

Supplementary material

Table 1 Supplementary Material. Percent difference between the initial anti-*Leishmania* IgG measure by ELISA and the repeated measure after storage at 20°C and 80°C for 6 months, 1 year, 1.5 years and 2.5 years. The %difference calculated is depicted as a function of the measurement at day zero.

| Animal status | 6 months (mean ±SD) | | 1 year (mean ±SD) | | | 1.5 years (mean ±SD) | | 2.5 years (mean ±SD) | |
| --- | --- | --- | --- | --- | --- | --- | --- | --- | --- |
|  | -20ºC | -80ºC | -20ºC | -80ºC | -20ºC | | -80ºC | -20ºC | -80ºC |
| Low positive | 9.46±12.03 | 5.37±10.69 | 0.85±11.07 | 4.21±15.99 | 3.66±13.95 | | 9.76±12.93 | 4.93±9.64 | 3.29±9.76 |
| Medium positive | 4.47±12.40 | -3.67±9.01 | 3.71±13.07 | -1.81±9.20 | -5.87±15.49 | | -4.55±3.62 | 5.87±29.79 | -7.11±8.91 |
| High Positive | 2.90±16.50 | 3.85±18.50 | -3.06±9.94 | -0.18±9.60 | -4.28±12.95 | | -3.94±10.75 | 11.02±42.86 | 1.95 ±18.37 |

SD=standard desviation

Figure 1 Supplementary Material. Percent difference between the initial anti-*Leishmania* IgG measure by ELISA and the repeated measure after storage at 20°C for 6 months, 1 year, 1.5 years and 2.5 years for low positive samples

Figure 2 Supplementary Material. Percent difference between the initial anti-*Leishmania* IgG measure by ELISA and the repeated measure after storage at 20°C for 6 months, 1 year, 1.5 years and 2.5 years for medium positive samples.

Figure 3 Supplementary Material. Percent difference between the initial anti-*Leishmania* IgG measure by ELISA and the repeated measure after storage at 20°C for 6 months, 1 year, 1.5 years and 2.5 years for high positive samples

Figure 4 Supplementary Material. Percent difference between the initial anti-*Leishmania* IgG measure by ELISA and the repeated measure after storage at 80°C for 6 months, 1 year, 1.5 years and 2.5 years for low positive samples.

Figure 5 Supplementary Material. Percent difference between the initial anti-*Leishmania* IgG measure by ELISA and the repeated measure after storage at 80°C for 6 months, 1 year, 1.5 years and 2.5 years for medium positive samples.

Figure 6 Supplementary Material. Percent difference between the initial anti-*Leishmania* IgG measure by ELISA and the repeated measure after storage at 80°C for 6 months, 1 year, 1.5 years and 2.5 years for high positive samples.
